# Tumor Lysis Syndrome in Solid Tumors: A Comprehensive Literature Review, New Insights, and Novel Strategies to Improve Outcomes

**DOI:** 10.7759/cureus.8355

**Published:** 2020-05-29

**Authors:** Dawood Findakly, Ross D Luther, Jue Wang

**Affiliations:** 1 Internal Medicine, Creighton University Arizona Health Education Alliance/Valleywise Health Medical Center, Phoenix, USA; 2 Urology, Madigan Army Medical Center, Tacoma, USA; 3 Genitourinary Oncology, Creighton University School of Medicine/University of Arizona Cancer Center at Dignity Health, Phoenix, USA

**Keywords:** tumor lysis syndrome, solid tumors, new insights, literature review, autopsy, disseminated intravascular coagulation (dic), cytokine storm syndrome

## Abstract

Tumor lysis syndrome (TLS) is a life-threatening oncological condition that is typically characterized by metabolic derangements that are often labeled as an acute kidney injury. The recent advancement in cancer treatment has led to the mounting rate of TLS in solid tumors that were previously rarely linked to this complication. Given that its prognosis is dismal, it is essential to increase recognition of this condition by describing more sensitive markers. Currently, the management of TLS is mainly supportive due to the lack of specific therapy targeting its specific pathology. This review aims to summarize the most recent literature on the underlying mechanism of TLS and the potential implications for novel TLS therapy.

## Introduction and background

Tumor lysis syndrome (TLS) is a life-threatening medical condition that can occur spontaneously or as a complication of cancer therapy for rapidly proliferating and chemo-sensitive malignancies such as acute lymphoblastic leukemia or high-grade lymphoma [1-3]. The devastating outcomes of TLS arise from rapid tumor cell lysis and the release of metabolites into the systemic circulation, therefore, leading to potentially fatal metabolic abnormalities, including hyperkalemia, hyperphosphatemia, hyperuricemia, and hypocalcemia [1].

A definitive TLS diagnosis requires one clinical and two laboratory criteria. Laboratory TLS is characterized by one of three scenarios. The first two are in the setting of a normal uric acid where a deranged phosphate and potassium levels, either exceed the upper limit of normal (ULN) or exhibit at least a 25% increase from normal levels. The third scenario is where uric acid exceeds the ULN or shows a 25% increase from baseline, including either elevated potassium or phosphate levels simultaneously. Coupled with these laboratory findings are many clinical criteria that often present as results of the underlying electrolyte abnormalities and accumulation of toxic metabolites within the blood. One key finding is creatinine elevation greater than or equal to 1.5 times the ULN, which may manifest as oliguria or anuria. Other common findings include cardiac arrhythmias and/or sudden death because of hyperkalemia [2-4].

Interestingly, acute kidney injury (AKI) may result from the deposition of uric acid or calcium phosphate crystals within renal tubules. AKI, besides hyperphosphatemia-driven hypocalcemia, can cause tetany and seizures [1,3,5,6]. Although reported in cases, physicians’ unscripted remarks labeling the forementioned renal consequences as an AKI is what contributes to the underestimated true incidence of TLS [3]. Given the high mortality of TLS, it is necessary to improve clinician awareness via the identification of more sensitive markers for this condition. This review aims to summarize the growing body of literature regarding TLS explicitly and to review relevant pathophysiological mechanisms to understand this syndrome better, therefore, ultimately, expand the depth of expertise and to pave the way to future evidence-based guidelines in order to find new standardized strategies for TLS prophylaxis and treatment.

## Review

Incidence of TLS in solid tumors

TLS typically occurs in patients with acute lymphoblastic leukemia (ALL) and non-Hodgkin lymphoma (NHL). The incidence of TLS comprises about 10% among patients undergoing remission-induction chemotherapy [7,8]. It is essential to recognize that the relatively recent and ongoing progress in cancer therapy has led to the rising incidence of TLS in solid tumors that were previously rarely thought to be associated [3]. The importance of this progress lies in the observation that mortality for TLS stemming from solid tumors is moderately high at 35% compared to 1.9% rate reported for patients with ALL and NHL [1,4,9-17].

Current understanding of TLS

An expanding body of research centered on the pathophysiology of TLS is utilized in order to understand its relation to clinical outcomes. Traditionally, AKI has been cited as the most common cause of death in patients with TLS due to solid tumors [10]. Risk factors for TLS are well-documented in the literature and are primarily driven by high tumor burden as defined by elevated lactate dehydrogenase, elevated white blood cells, bulky disease, high proliferation rate, and organ metastases [18-20]. Moreover, advanced age predisposes to TLS, as it correlates with a reduction in decreased glomerular filtration rate and heightened susceptibility to dehydration [3].

New insight of TLS

Animal Models: Understanding the Pathophysiology of TLS

Despite the aforementioned predisposing factors, animal models of TLS have shown resilience in some mice with heavy tumor burdens, suggesting that heavy tumor burden itself is necessary but not sufficient to cause TLS. This has notably prompted efforts to investigate further pathophysiologic mechanisms of tissue damage that may explain the high mortality of TLS [21-25]. For example, studies in mice revealed that disseminated microemboli composed of debris from lysed tumor cells prompted mechanical micro-obstruction of multiorgan capillary beds, including the kidney tubules, brain, lungs, and elsewhere. These processes ultimately resulted in widespread tissue ischemia, necrosis, and early death [21,22]. This finding proposes an alternative to the classic picture of TLS-driven AKI through renal tubular crystal deposition. On the other hand, this may suggest several potential mechanisms for developing AKI. This further proposed a possible synergistic process through a microemboli-driven decreased renal perfusion resulting in oliguria and tissue hypoxia, which subsequently promote crystal deposition and necrosis of the renal tubular epithelium (Table 1). Finally, the dissemination of microemboli may underlie the other nonspecific clinical signs associated with TLS in humans, such as respiratory distress and mental status changes.

**Table 1:** Review of TLS in animal model findings RCT: Randomized controlled trial; TLS: Tumor lysis syndrome; STLS: Spontaneous TLS; LN: Lymph nodes; NNC: Necrotic neoplastic cells; NM: Not mentioned; N/A: Not applicable; MOF: Multi-organ failure; RF: Renal failure; DIC: Disseminated intravascular coagulation; Ref: Reference.

Author	Experimental Model	Treatment Type vs. Spontaneous	Cancer Pathology	Metastases	Microemboli Composition	Post-Mortem Pathology	Clinical Presentation	Ref
Lovelace et al.	Case Report: 10-month-old female sentinel mouse	STLS	Leukemic lymphoma	Cranial, liver, spleen, and mediastinal, submandibular, and mesenteric LN	Mixed eosinophilic and basophilic (aggregated chromatin) NNC	Embolic vascular occlusions within the lungs, kidneys, brain, liver	Seizure	[21]
Vogel et al.	RCT: p53^+/-^ mice (50% female, 50% male)	STLS	Disseminated lymphoblastic lymphoma and leukemia, bladder neoplasia	Spleen, liver, multiple LN chains	Mixed basophilic (aggregated chromatin) and eosinophilic NNC	Microembolic occlusions within liver, lung, brain	NM	[22]
Calia et al.	Case Report: 2-year-old female domestic short hair cat, feline leukemia virus-positive	Chemotherapy: IP injection L-asparaginase (400 mg/kg), prednisone (5 mg PO bid) Radiation: Cobalt 60 teletherapy, 800 cGy	Lymphoma	Brain, and mediastinal mass	N/A	N/A	Respiratory failure, arrhythmia	[23]
LaCarrubba et al.	Case Report: 11-year-old mare (female horse)	STLS	Peritoneal mesothelioma	Direct extension into thoracic cavity	High-grade neoplastic cells staining positive for cytokeratin and vimentin	Myxomatous degeneration of ascending aorta, main pulmonary artery	SIRS, MOF, arrhythmia, RF	[24]
Mylonakis et al.	Case Report: 5-year-old female German Shepherd dog	Chemotherapy 1^st^ protocol: Cyclophosphamide, vincristine, cytosine, arabinoside, prednisolone. Chemotherapy 2^nd^ protocol: Vincristine, L-asparaginase, prednisolone, cyclophosphamide, doxorubicin.	B-cell multicentric lymphoma	N/A	N/A	N/A	MOF, DIC, RF	[25]

Human Autopsy Studies

Several documented case reports of TLS in humans appear to echo animal model findings, as microemboli appear to play a direct role in widespread tissue damage [20-34]. The demographics, clinicopathologic features, autopsy findings, and outcomes are summarized in Table 2. Takeuchi et al. reported rapid decompensation within 40 hours of admission in a patient with spontaneous TLS secondary to malignant melanoma. Interestingly, the autopsy revealed a key finding of massive necrosis of normal hepatocytes and melanoma cells within the liver as well as disseminated tumor thrombi in the portal system. Additionally, they found extensive necrosis within a large pulmonary tumor [26]. Moreover, similar findings were reported by Kearney et al. where an autopsy in a patient with STLS revealed extensive tumor necrosis, widespread tumor thrombi in portal venous branches, pulmonary tumor deposits, and near-total replacement of lymph nodes with tumor and necrosis [27]. These findings indicate that TLS stemming from a solid tumor likely induces necrosis within both tumor and non-tumor cells that are distant from the tissue of origin. Ito et al. reported a patient with TLS following docetaxel and carboplatin treatment for recurrent endometrial cancer. This patient developed classic laboratory abnormalities for TLS, AKI, in conjunction with sudden onset chest pain, hypoxia, and respiratory distress 19 hours after initiation of chemotherapy, and the autopsy, thereafter, showed pulmonary tumor embolism [28].

**Table 2:** Review of TLS in human autopsy findings M: Male; F: Female; TLS: Tumor lysis syndrome; STLS: Spontaneous TLS; MOF: Multi-organ failure; SIRS: Systemic inflammatory response syndrome; RF: Renal failure; BM: Bone marrow; LN: Lymph nodes; DIC: Disseminated intravascular coagulation; PE: Pulmonary embolism; ICH: Intracranial hemorrhage; VAC: Vincristine, actinomycin D, cyclophosphamide; RCC: Renal cell carcinoma; cGY: Centigray.

Author	Age/Gender	Treatment-Driven vs. Spontaneous	Primary Tumor Histology	Metastatic Site(s)	Clinical Features	Outcomes	Pathologic Findings	Ref
Allen et al.	55/M	STLS	RCC, rhabdoid features	Liver	SIRS, RF, MOF	Death	MOF	[20]
Takeuchi et al.	62/M	STLS	Stage IV Melanoma	Liver, lungs, vertebrae	MOF, arrhythmia	Death	Massive hepatocyte necrosis and pulmonary tumor cells, disseminated tumor thrombi in the portal system.	[26]
Kearney et al.	47/F	STLS	Stage IV sigmoid adenocarcinoma	Liver	SIRS, RF, MOF	Death	Extensive necrosis and widespread tumor thrombi in portal venous system, tumor deposits in lungs, near total replacement of LN with tumor and necrosis	[27]
Ito et al.	63/F	Tri-weekly docetaxel, carboplatin	Recurrent stage IV uterine serous carcinoma	Lung, kidney, spine, pelvis, aorta	SIRS, MOF, arrhythmia, respiratory failure, RF	Death	Pulmonary tumor embolus	[28]
Bien	14/M	STLS	Embryonal rhabdomyosarcoma, not detected	BM, pleura, peritoneum, LN	DIC	Survived	DIC, pulmonary hemorrhage	[29]
Bien	14/F	STLS	Parietal rhabdomyosarcoma	BM	DIC, PE	Survived	DIC, PE, ICH	[29]
Watanabe and Tanaka	16/M	STLS and treatment-driven (VAC)	Alveolar rhabdomyosarcoma, Prostate	BM, LN, bladder wall	DIC, RF	Survived	DIC, bladder hemorrhage	[30]
Nguyen and Ticona	72/M	STLS	Stage IV prostate carcinoma	Bone	SIRS, arrhythmia, DIC, RF	Survived	DIC, retro-peritoneal hemorrhage	[31]
Teh and Tsoi	54/M	Carboplatin, etoposide, direct trauma (biopsy)	Neuroendocrine carcinoma, pancreatic primary	Liver	DIC, RF, MOF	Death	RF, Liver failure, DIC	[32]
Liu and Monaco	43/M	Radiation therapy (2,000 cGy) to the stomach	Stage IV gastric adenocarcinoma	Liver, liver, stomach, LN, gall bladder	SIRS, RF, MOF	Death	Extensive necrosis in the metastatic sites	[33]
Barton	57/F	Cyclophosphamide, methotrexate, 5-fluorouracil	Metastatic infiltrating ductal breast carcinoma	Liver, pleurae, lungs, chest wall	SIRS, RF	Death	Extensive lymphangitic carcinoma of the lungs, tumor infiltrating the pleurae, acute necrosis of hepatic metastasis	[34]

Understanding Disseminated Intravascular Coagulation (DIC) and TLS

The association between DIC and neoplasms is well-known and is not uncommon. DIC is estimated to present in 15-20% of patients with acute leukemia, specifically the acute promyelocytic leukemia (APL) variant, and 7-15% of patients with solid metastatic tumors [23]. Several reports in the literature support the hypothesis that TLS and DIC were co-occurring, with one seemingly providing a nidus for the other and contributing to a rapid synergistic decline in patients' hemodynamic status. Watanabe and Tanaka presented a case of a 16-year-old Japanese male who developed prostate rhabdomyosarcoma with multiple bony metastases and found to have laboratory TLS findings upon initial evaluation. The patient subsequently developed DIC just one week following biopsy. Rasburicase administered on day 1 of chemotherapy initiation. On day 3 of treatment, the patient developed a worsening of his DIC secondary to tumor lysis, and the patient ultimately recovered due to early recognition of TLS [30]. A similar instance of concurrent TLS and DIC reported in a case of untreated prostate cancer with widely metastatic bony metastases [31]. In this case, the patient developed spontaneous TLS with subsequent worsening of DIC but fully recovered due to early initiation of rasburicase and appropriate supportive care. Another case of concurrent TLS and DIC was reported in a patient with metastatic neuroendocrine carcinoma following manipulation by core biopsy and then again following the initiation of cytotoxic chemotherapy [32]. The significance of these presentations lies in the common pathogenesis as pro-inflammatory cytokines mediate both TLS and DIC.

The Role of Hypercytokinemia in TLS

Further complicating the management of TLS, clinicians must be aware of the potential of hypercytokinemia, or cytokine storm as a consequence of cancer treatment and how this may necessitate alternate therapy modalities to address systemic inflammatory response syndrome (SIRS) and multi-organ failure (MOF). Hypercytokinemia involves the release of intracellular cytokines (e.g., TNF-alpha, IL-6, IL-10), which induces SIRS leading to MOF and hemodynamic instability marked by hypotension and tachycardia [35-38]. Hsu et al. and Schonbohn et al. found increased blood levels of IL-6, IL-8, IL-1, and TNF-alpha following single courses of chemotherapy for acute myelogenous leukemia (AML) [39,40]. Born out of these findings came the hypothesis that TLS may be the driver for cytokine storm, as rapid cell lysis causes the release of these pro-inflammatory cytokines which collectively induce nitric oxide-mediated vasodilation and systemic inflammation. Interestingly, IL-1, IL-6, and TNF-alpha may contribute to DIC via the induction of the hepatic release of clotting factors (e.g., fibrinogen) and other acute-phase reactants (e.g., C-reactive protein), culminating in SIRS and MOF. For instance, Nakamura et al. observed four patients with baseline renal dysfunction who developed TLS within two days of chemotherapy initiation, 100% of which progressed to kidney failure and 50% to MOF. Key findings from this study include: (1) elevated serum IL-6 levels in 100% of cases with a blood level tended to be proportional to the increase in the numbers of organs failing; (2) the serum levels of other cytokines (TNF-alpha, IL-8 and IL-10) were elevated in one of the cases; and (3) all patients survived following 3-7 days of polymethylmethacrylate membrane continuous hemodiafiltration (PMMA-CHDF) for cytokine removal [36]. Similarly, Soares et al. observed five patients with confirmed TLS via both spontaneous (n = 2) and chemotherapy-driven mechanisms (n = 3) who all fulfilled SIRS criteria and had at last three distinct organ dysfunctions (e.g., respiratory, renal, hepatic). All patients required ventilator support, and 80% treated with renal replacement therapy. Among those patients, 40% ultimately died, with 2/3 of the surviving patients regaining full renal function [37].

The question remains as to the temporal relationship between TLS and cytokine storm, but the comorbidity and interplay of processes cannot be ignored. Hyperuricemia itself, a key marker for TLS, may act as a free-radical scavenger and increase in response to oxidative stress. Additionally, urate crystals are known to activate inflammatory pathways and induce a systemic inflammatory response [30].

## Conclusions

TLS is an oncological emergency that challenges providers to imply hypervigilance in monitoring fluid status, hemodynamics, clotting factors, electrolytes, and cytokines in order to expeditiously identify, respond, and, ultimately, prevent many devastating downstream consequences. Based on the latest studies, TLS-associated DIC as well as hypercytokinemia are novel therapeutic targets in treating this potentially fatal condition. We are in the process of developing a clinical study evaluating this novel strategy.
